# Prevalence, Associated Risk Factors, and the Impact of Malaria on Anemia in Pregnant Women Attending ANC in North Gondar Zone, Northern Ethiopia

**DOI:** 10.1155/bmri/4046408

**Published:** 2026-04-18

**Authors:** Gebre Ayanaw Alula, Tilahun Endale, Gebeyehu Yibeltie

**Affiliations:** ^1^ Department of Biology, College of Natural and Computational Science, Debark University, Debark, Ethiopia, dku.edu.et

**Keywords:** anemia, Ethiopia, malaria, North Gondar, pregnancy, risk factors

## Abstract

Malaria during pregnancy continues to be a major public health concern in sub‐Saharan Africa, contributing to maternal anemia and adverse pregnancy outcomes. This study is aimed at assessing the prevalence of malaria, its associated risk factors, and its impact on anemia among pregnant women attending antenatal care in North Gondar Zone, Northern Ethiopia. A facility‐based cross‐sectional study was conducted with 308 pregnant women selected through systematic random sampling. Sociodemographic, environmental, and behavioral data were collected using structured questionnaires. Malaria infection was diagnosed with Giemsa‐stained blood smears, and hemoglobin concentration was measured using a Hemocue Hb 201+ analyzer. Data analysis was performed using SPSS Version 26, with bivariate and multivariate logistic regression used to identify factors associated with malaria. The impact of malaria on anemia was assessed using adjusted odds ratios (AORs) with 95% confidence intervals (CIs). The overall malaria prevalence was 13.6%, with higher odds observed among women aged ≥ 40 years (AOR = 4.8; 95% CI: 1.4–16.0), rural residents (AOR = 2.8; 95% CI: 1.2–6.5), and women with low education (AOR = 2.6; 95% CI: 1.0–6.7). Environmental and behavioral factors, including living within 1 km of water bodies (AOR = 3.0; 95% CI: 1.4–6.5), unscreened houses (AOR = 3.6; 95% CI: 1.6–8.2), and inconsistent or nonuse of insecticide‐treated nets (AOR = 7.2; 95% CI: 2.9–18.0), were also significant. Among malaria‐infected women, 40% were anemic, and malaria significantly increased the risk of anemia. These findings highlight malaria as a key contributor to maternal anemia and underscore the importance of targeted interventions, including consistent ITN use, environmental management, community education, and integrated malaria–anemia screening during antenatal care, to improve maternal health in endemic areas.

## 1. Introduction

Malaria continues to be a major public health challenge worldwide, particularly in sub‐Saharan Africa, where transmission remains high despite decades of control efforts [1]. Each year, an estimated 25 million pregnant women in the region are at risk of Plasmodium infection, which can lead to serious maternal and neonatal complications, including maternal anemia, low birth weight, preterm delivery, and perinatal mortality [2, 3]. In Ethiopia, Plasmodium falciparum and Plasmodium vivax are the dominant malaria species, with P. falciparum responsible for the majority of cases nationwide [4]. Pregnancy weakens the immune system to some extent, making women more susceptible to malaria infection and its severe consequences, such as placental parasitemia and intrauterine growth restriction [5].

Anemia during pregnancy, defined as hemoglobin below 11 g/dL, remains a major concern in low‐ and middle‐income countries due to its association with maternal mortality, postpartum hemorrhage, and adverse neonatal outcomes [6, 7]. While anemia has multiple causes, including nutritional deficiencies and chronic diseases, malaria remains a leading contributor in endemic areas due to hemolysis of infected and uninfected erythrocytes and suppression of erythropoiesis [8].

In Ethiopia, malaria transmission is highly variable, influenced by altitude, rainfall, and other environmental factors. About 75% of the country′s landmass is considered malarious, placing a large portion of the population at risk (National Malaria Indicator Survey) [9]. Despite national strategies such as insecticide‐treated bed nets (ITNs), indoor residual spraying (IRS), and prompt case management, persistent transmission and localized outbreaks remain a challenge [10]. Malaria prevalence among pregnant women also varies widely, with studies reporting figures such as 24.1% in the West Guji Zone [11], reflecting differences in transmission intensity, preventive coverage, and seasonal factors.

Previous studies conducted in Ethiopia have examined malaria prevalence among pregnant women, associated risk factors such as ITN utilization, gravidity, and gestational age, as well as the magnitude of anemia in pregnancy as a separate public health concern [4, 5, 11]. Some investigations have also reported associations between malaria infection and reduced hemoglobin levels [8]. However, most of these studies have primarily focused on either malaria or anemia independently, or have assessed their relationship using limited analytical approaches, often without controlling for important confounding variables such as nutritional status, socioeconomic conditions, or access to antenatal care (ANC) services. In addition, many studies have relied on cross‐sectional descriptive analyses without adequately exploring the strength and direction of association between malaria and anemia using robust statistical models [2, 6].

Furthermore, the majority of existing evidence in Ethiopia originates from referral hospitals or urban‐based health facilities, which typically serve populations with relatively better healthcare access, diagnostic capacity, and malaria prevention coverage [4, 11]. These settings may not accurately represent the epidemiological conditions in primary hospitals, where healthcare resources are often limited, laboratory diagnostic capacity may be constrained, and patients are more likely to come from rural or semiurban communities with higher exposure to malaria risk factors [4]. Consequently, findings from referral or urban hospitals may underestimate the true burden of malaria and anemia, as well as their interaction, in underserved populations [7, 10].

Despite substantial research on malaria or anemia separately, evidence remains limited on their interrelationship among pregnant women in primary healthcare settings [12]. This indicates a critical knowledge gap regarding how malaria contributes to anemia under real‐world conditions in primary hospitals, where variations in prevention practices, delayed healthcare seeking, and environmental exposure may significantly influence both conditions. Many prior studies have been conducted in referral hospitals or urban centers, potentially overlooking the epidemiological context of women attending primary hospitals that serve predominantly rural and semiurban populations with limited access to preventive tools and early diagnosis [13, 14].

Understanding the prevalence of malaria, its associated risk factors, and its impact on anemia among pregnant women attending ANC at primary hospitals is therefore essential. Identifying modifiable risk factors such as inconsistent ITN use, late initiation of ANC, gravidity, gestational age, socioeconomic status, and nutritional conditions can inform targeted strategies for prevention and control [15]. Moreover, quantifying the relationship between malaria and anemia will contribute to integrated maternal health interventions that address both infectious and hematological conditions.

Addressing this gap is crucial for improving maternal and neonatal health outcomes in malaria‐endemic areas. The findings of this study can assist local health authorities and policymakers in strengthening malaria prevention during pregnancy, improving anemia screening and management, and enhancing ANC services at the primary healthcare level. Therefore, this study is aimed at determining the prevalence of malaria, identifying associated risk factors, and assessing its impact on anemia among pregnant women attending ANC at primary and general hospitals in North Gondar Zone, Northwest Ethiopia.

## 2. Methodology

### 2.1. Study Area

The study was conducted in selected hospitals within North Gondar Zone, Amhara Regional State, Northwest Ethiopia, located about 830 km northwest of Addis Ababa and 103 km from Gondar. The area lies at 13°8 ^′^ N latitude and 37°54 ^′^ E longitude, with an altitude of 2850 m above sea level. It receives 1900–2400 mm of annual rainfall, and the mean annual temperature ranges from 12°C to 20°C.

North Gondar Zone has a population of 905,680 (425,846 men and 479,834 women) and comprises mainly rural and semiurban communities. Seasonal malaria transmission is influenced by rainfall, altitude, stagnant water, and irrigation practices. Hospitals in the zone provide ANC and other maternal health services to women who are particularly vulnerable to malaria and anemia due to seasonal transmission, limited preventive measures, and nutritional challenges. These conditions make the hospitals suitable for assessing malaria prevalence, associated risk factors, and its impact on anemia among pregnant women.

### 2.2. Study Design

A health facility–based cross‐sectional study was conducted to assess the prevalence of malaria, associated risk factors, and its impact on anemia among pregnant women attending ANC services in selected hospitals of North Gondar Zone. Data on sociodemographic characteristics, environmental exposures, and clinical outcomes were collected at a single point in time from each participant. This design allowed for the simultaneous assessment of malaria infection, anemia status, and potential contributing factors within the study population.

### 2.3. Study Population

The study population consists of pregnant women attending ANC services at the selected hospitals in North Gondar Zone during the study period. These women represent the population at risk of malaria and anemia in the zone and are the focus for assessing prevalence, associated risk factors, and health outcomes.

### 2.4. Inclusion and Exclusion Criteria

#### 2.4.1. Inclusion Criteria

All pregnant women attending ANC services at the selected hospitals during the study period who were willing to participate and provided informed consent were included in the study.

#### 2.4.2. Exclusion Criteria

Pregnant women who were severely ill and unable to respond to the questionnaire, those who had received antimalarial treatment within the previous 2 weeks, and those with incomplete data were excluded from the study.

### 2.5. Sample Size Determination

The required sample size was determined using the single population proportion formula.

n=Z2∗p 1−pd2

where n is the required sample size, Z^2^ is the standard normal value at 95% confidence level (1.96), p is the estimated prevalence of malaria among pregnant women (23.9% = 0.239), and d is the margin of error (0.05)

After the calculation, the initial sample size was 280 participants. To compensate for a potential 10% nonresponse rate, the sample size was adjusted to 308 participants, which was considered the final sample size for the study. The prevalence of malaria (23.9%) was obtained from a previous study conducted in the wetlands of Bahir Dar Zuria District, Northwestern Ethiopia [16].

### 2.6. Sampling Technique

Participants were selected using a systematic random sampling technique. The total sample size (308) was proportionally allocated across the study period based on the average daily ANC attendance in the selected hospitals. The sampling interval (k) was calculated by dividing the estimated total number of ANC attendees during the data collection period (N) by the required sample size (n).

The first participant was selected randomly using a lottery method among the first k attendees, and thereafter, every k‐th eligible pregnant woman was included. If a selected participant did not meet the inclusion criteria or declined to participate, the next eligible participant was recruited to maintain the sampling interval.

### 2.7. Data Collection

#### 2.7.1. Questionnaire Data

A structured questionnaire, adapted from relevant literature, was used to collect sociodemographic, obstetric, environmental, and behavioral data.

Operational definitions of key variables were established to ensure consistency:

• **ITN use:** sleeping under an ITN the night before the survey (yes/no)
• **Outdoor nighttime activity:** staying outdoors for ≥ 2 h between 6:00 PM and 6:00 AM
• **Malaria history:** self‐reported malaria infection within the past 12 months
• **Knowledge about malaria:** assessed using a set of structured questions and categorized as good or poor based on the mean score

The questionnaire was pretested on 5% of pregnant women at a nearby health facility. Based on pretest findings, unclear questions were revised, ambiguous wording was simplified, and the sequence of questions was adjusted to improve clarity and flow. Reliability and consistency of responses were also checked during pretesting.

The questionnaire was prepared in English and translated into Amharic for data collection. Back‐translation into English was performed to ensure accuracy, and cultural adaptations were made to improve comprehension in the local context.

Data collection was carried out by two trained research assistants and one laboratory technician under the supervision of the principal investigator. All data collectors were trained on interview techniques, ethical considerations, and standardized procedures to minimize bias and ensure high‐quality data collection.

#### 2.7.2. Laboratory Data

Laboratory investigations were conducted to assess malaria infection and hemoglobin concentration. Malaria diagnosis was performed using Giemsa‐stained thick and thin blood smears prepared from finger‐prick blood samples, following WHO standard protocols. Thick smears were used to determine parasite density, whereas thin smears were used for Plasmodium species identification. Parasite density was calculated as the number of parasites per microliter of blood, assuming a standard white blood cell count of 8000/*μ*L.

Hemoglobin concentration was measured using a Hemocue Hb 201+ analyzer following the manufacturer′s instructions. Capillary blood samples were collected via finger‐prick, and hemoglobin levels were determined immediately.

Malaria infection status was defined as the presence of Plasmodium parasites on microscopy (positive/negative). Anemia was defined according to WHO criteria (hemoglobin < 11 g/dL) and classified as mild (10–10.9), moderate (7–9.9), and severe (< 7 g/dL). Gestational age was categorized into first trimester (< 12), second trimester (13–27), and third trimester (≥ 28 weeks).

All slides were examined independently by two experienced microscopists, and discrepancies were resolved by a senior microscopist. Regular calibration of the Hemocue device was performed to maintain accuracy.

### 2.8. Data Analysis

Data were entered into EpiData Version 4.6 and analyzed using SPSS Version 26. Descriptive statistics, including frequencies, percentages, means, and standard deviations, were used to summarize sociodemographic and clinical characteristics. The prevalence of malaria and anemia was calculated with 95% confidence intervals. Bivariate logistic regression was used to identify potential risk factors for malaria infection, and variables with p < 0.25 were included in multivariate logistic regression to control for confounding. The impact of malaria on anemia was assessed using adjusted odds ratios and 95% confidence intervals, accounting for age, gravidity, gestational age, and ITN use, with statistical significance set at p < 0.05.

## 3. Results

### 3.1. Sociodemographic and Obstetric Characteristics of Study Participants

A total of 308 pregnant women attending ANC services in selected hospitals of North Gondar Zone participated in the study. The majority were aged 30–34 years (31.8%), followed by 25–29 years (27.6%). Most participants resided in rural areas (60.7%) and were married (88.6%). Regarding education, 40.3% had completed elementary school, whereas 20.5% were illiterate. Over half of the women (53.6%) reported a monthly income between 2000–5000 Ethiopian birr (≈ USD 15–35). Private sector employment was the most common occupation (54.2%), followed by NGO work (28.2%) and government employment (17.5%).

Regarding obstetric factors, 41.6% were primigravida, 36.7% were secundigravida, and 21.7% were multigravida. Most participants (63.0%) were in their second trimester at the time of data collection. These sociodemographic and obstetric characteristics are summarized in Table 1.

**Table 1:** Sociodemographic characteristics of pregnant women in North Gondar Zone, Ethiopia, 2025 (n = 308).

Variables	Frequency (*n*)	Percentage (%)
Age category		
15–19 years	19	6.2%
20–24 years	67	21.7%
25–29 years	85	27.6%
30–34 years	98	31.8%
35–39 years	23	7.5%
≥ 40 years	16	5.2%
Residence		
Urban	121	39.3%
Rural	187	60.7%
Marital status		
Single	14	4.5%
Married	273	88.6%
Divorced	13	4.2%
Widowed	8	2.6%
Level of education		
Illiterate	63	20.5%
Elementary	124	40.3%
High school	89	28.9%
College and above	32	10.4%
Monthly income		
Below 2000 birr (≈ USD 15)	51	16.6%
2000–5000 birr (≈ USD 15–35)	165	53.6%
Above 5000 birr (≈ USD 35)	92	29.9%
Occupation		
Private	167	54.2%
Government employee	54	17.5%
Nongovernment employee	87	28.2%
Gravidity		
Primigravida	128	41.6%
Secundigravida	113	36.7%
Multigravida	67	21.7%
Gestational age		
First trimester	55	17.9%
Second trimester	194	63.0%
Third trimester	59	19.1%

### 3.2. Malaria Prevalence and Sociodemographic Predictors

The overall malaria prevalence among pregnant women was 13.6% (42/308; 95% CI: 10.0%–17.7%). P. falciparum accounted for 85.7% of infections, whereas P. vivax accounted for 14.3%. Older age (≥ 40 years), rural residence, and low educational level were significant predictors of malaria infection. Although women with lower monthly income < 2000 ETB (≈ USD less than 15) showed higher prevalence (19.6%), this association was not statistically significant after adjustment. Marital status, occupation, gravidity, and gestational age were not independently associated with malaria. Detailed analysis of malaria prevalence and its association with sociodemographic factors is presented in Table 2.

**Table 2:** Malaria prevalence and associated sociodemographic factors in pregnant women in North Gondar Zone, Ethiopia, 2025 (n = 308).

Variables	Frequency (*n*)	Prevalence	COR (95% CI)	*p*	AOR (95% CI)	*p*
Age						
15–19 years	19 (6.2%)	3 (15.8)	2.4 (0.7–8.1)	0.153	2.1 (0.6–7.5)	0.206
20–24 years	67 (21.8%)	8 (11.9)	1.8 (0.7–4.8)	0.228	1.6 (0.6–4.3)	0.332
25–29 years	85 (27.6%)	10 (11.8)	1.7 (0.7–4.0)	0.210	1.5 (0.6–3.7)	0.357
30–34 years	98 (31.8%)	12 (12.2)	1.8 (0.8–3.9)	0.182	1.6 (0.7–3.6)	0.283
35–39 years	23 (7.5%)	4 (17.4)	2.6 (0.8–8.2)	0.116	2.3 (0.7–7.5)	0.146
≥ 40 years	16 (5.2%)	5 (31.3)	5.5 (1.7–17.7)	0.005∗	4.8 (1.4–16.0)	0.011∗
Residence						
Urban	121 (39.3%)	8 (6.6)	1	—	1	—
Rural	187 (60.7%)	34 (18.2)	3.2 (1.4–7.2)	0.015∗	2.8 (1.2–6.5)	0.032∗
Marital status						
Single	14 (4.5%)	1 (7.1)	0.8 (0.1–5.2)	0.821	0.9 (0.1–6.1)	0.906
Married	273 (88.6%)	39 (14.3)	1	—	1	—
Divorced	13 (4.2%)	1 (7.7)	0.9 (0.1–6.3)	0.883	0.8 (0.1–5.8)	0.851
Widowed	8 (2.6%)	1 (12.5)	0.9 (0.1–7.6)	0.921	0.9 (0.1–7.0)	0.914
Level of education						
Illiterate	63 (20.5%)	13 (20.6)	3.0 (1.2–7.4)	0.026∗	2.6 (1.0–6.7)	0.041∗
Elementary	124 (40.3%)	17 (13.7)	2.0 (0.9–4.5)	0.083	1.8 (0.8–4.2)	0.123
High school	89 (28.9%)	9 (10.1)	1.5 (0.6–3.6)	0.377	1.3 (0.5–3.3)	0.561
College and above	32 (10.4%)	3 (9.4)	1	—	1	—
Monthly income						
Below 2000 birr (≈ USD 15)	51 (16.6%)	10 (19.6)	2.8 (1.0–7.7)	0.045∗	2.4 (0.9–6.4)	0.076
2000–5000 birr (≈ USD 15–35)	165 (53.6%)	22 (13.3)	1.8 (0.8–4.1)	0.120	1.6 (0.7–3.8)	0.218
Above 5000 birr (≈ USD 35)	92 (29.9%)	10 (10.9)	1	—	1	—
Occupation						
Private	167 (54.2%)	20 (12.0)	1.1 (0.5–2.3)	0.758	1.0 (0.5–2.2)	0.974
Government employee	54 (17.5%)	7 (13.0)	1.2 (0.4–3.2)	0.731	1.1 (0.4–2.9)	0.863
Nongovernment employee	87 (28.2%)	15 (17.2)	1.5 (0.7–3.2)	0.273	1.4 (0.6–3.1)	0.342

Note: Asterisk “∗” denotes statistically significant p value.

### 3.3. Environmental and Behavioral Determinants of Malaria

Malaria prevalence among pregnant women was strongly influenced by environmental and behavioral factors (Table 3). Women living within 1 km of water bodies had the highest prevalence (20.7%) and were significantly more likely to be infected than those living more than 2 km away (AOR = 3.0, 95% CI: 1.4–6.5, p = 0.004). Housing type also affected risk, with residents of mud/thatched houses showing higher prevalence (14.1%) compared with those in brick or concrete houses (AOR = 2.4, 95% CI: 1.1–5.2, p = 0.015). Similarly, households without screened windows and doors had significantly higher malaria prevalence than screened households (AOR = 3.6, 95% CI: 1.6–8.2, p = 0.002).

**Table 3:** Environmental and behavioral determinants of malaria among pregnant women in North Gondar Zone, Ethiopia, 2025 (n = 308).

Characteristic	Frequency (*n*)	Prevalence (*n*/%)	COR (95% CI)	*p*	AOR (95% CI)	*p*
Proximity to water bodies						
< 1 km	92 (29.9)	19 (20.7%)	3.5 (1.6–7.8)	0.002∗	3.0 (1.4–6.5)	0.004∗
1–2 km	121 (39.3)	12 (9.9%)	1.4 (0.6–3.3)	0.433	1.3 (0.5–3.1)	0.552
> 2 km	95 (30.8)	11 (11.6%)	1	—	1	—
Housing type						
Mud/thatched	128 (41.6)	18 (14.1%)	2.8 (1.3–6.4)	0.007∗	2.4 (1.1–5.2)	0.015∗
Wood/iron	102 (33.1)	13 (12.7%)	1.7 (0.7–3.8)	0.229	1.6 (0.7–3.7)	0.263
Brick/concrete	78 (25.3)	11 (14.1%)	1	—	1	—
Window/door screening						
Screened	141 (45.8)	8 (5.7%)	1	—	1	—
Unscreened	167 (54.2)	34 (20.4%)	4.0 (1.8–8.6)	0.001∗	3.6 (1.6–8.2)	0.002∗
Land use/vegetation						
Agricultural (irrigated)	111 (36.0)	19 (17.1%)	3.2 (1.4–7.2)	0.082	2.9 (1.2–6.8)	0.126
Bushy/forest	99 (32.1)	12 (12.1%)	1.9 (0.8–4.3)	0.154	1.8 (0.7–4.1)	0.181
Urban/cleared	98 (31.8)	11 (11.2%)	1	—	1	—
Seasonal period						
Rainy season	101 (32.8)	20 (19.8%)	3.8 (1.6–8.8)	0.002∗	3.5 (1.5–8.5)	0.003∗
Postrainy	107 (34.7)	12 (11.2%)	2.1 (0.9–5.1)	0.125	2.0 (0.8–4.7)	0.152
Dry season	100 (32.5)	10 (10.0%)	1	—	1	—
Use of preventive measures						
ITN consistently used	119 (38.6)	5 (4.2%)	1	—	1	—
ITN occasionally used	112 (36.4)	14 (12.5%)	4.0 (1.7–9.5)	0.001∗	3.6 (1.5–8.6)	0.003∗
No ITN	77 (25.0)	23 (29.9%)	8.0 (3.3–19.4)	< 0.001∗	7.2 (2.9–18.2)	< 0.001∗
Outdoor nighttime activity						
Frequent	91 (29.5)	17 (18.7%)	3.5 (1.5–8.6)	0.125	3.0 (1.3–6.7)	0.154
Occasional	109 (35.4)	15 (13.8%)	1.9 (0.8–4.2)	0.221	1.8 (0.7–4.3)	0.250
Rare	108 (35.1)	10 (9.3%)	1	—	1	—
Health‐seeking behavior						
Prompt treatment	141 (45.8)	11 (7.8%)	1	—	1	—
Delayed treatment	109 (35.4)	22 (20.2%)	3.0 (1.4–6.2)	0.123	2.7 (1.2–6.1)	0.156
Traditional remedies	58 (18.8)	9 (12.0%)	1.5 (0.5–4.3)	0.482	1.4 (0.5–4.4)	0.521
Knowledge/awareness						
High	111 (36.0)	6 (5.4%)	1	—	1	—
Moderate	97 (31.5)	13 (13.4%)	2.4 (1.0–5.7)	0.128	2.2 (0.9–5.3)	0.154
Low	100 (32.5)	23 (23.0%)	5.6 (2.3–13.5)	0.081	5.0 (2.0–12.6)	0.103

Note: Asterisk “∗” denotes statistically significant p value.

Land use patterns and seasonal variation were also important determinants. Women living in agricultural or irrigated areas had a higher prevalence (17.1%), and those surveyed during the rainy season were more likely to be infected than in the dry season (AOR = 3.5, 95% CI: 1.5–8.0, p = 0.003). Preventive measures were strongly protective; consistent use of ITNs was associated with the lowest prevalence (4.2%), whereas occasional use (12.5%) and nonuse (29.9%) significantly increased the risk of malaria (AOR = 3.6, 95% CI: 1.5–8.6, p = 0.003 and AOR = 7.2, 95% CI: 2.9–18.0, p < 0.001, respectively).

### 3.4. Anemia in Malaria‐Infected Pregnant Women

Among the 42 malaria‐positive women, 17 (40%) were anemic, indicating a substantial burden of anemia among infected women. The mean hemoglobin among malaria‐positive women was 10.4 ± 1.2 g/dL, compared with 11.3 ± 0.9 g/dL among malaria‐negative women (p < 0.001), suggesting a significant impact of malaria on hemoglobin levels.

### 3.5. Anemia and Its Determinants Among Pregnant Women

The overall prevalence of anemia among the 308 pregnant women was 5.5%. Maternal age and residence were not significantly associated with anemia. Women living within 1 km of a water body had higher odds of anemia compared with those living farther away (AOR = 2.8; 95% CI: 0.9–7.6, p = 0.03). Nonuse of ITNs was also a significant risk factor (AOR = 3.1; 95% CI: 0.9–10.5, p = 0.04). Low knowledge about anemia increased the risk of anemia (AOR = 3.2; 95% CI: 1.0–10.5, p = 0.04). Primigravida women were significantly more likely to be anemic than multigravida women (AOR = 3.0; 95% CI: 1.2–7.4, p = 0.02). Gestational age was not significantly associated with anemia. The determinants of anemia among pregnant women are summarized in Table 4.

**Table 4:** Determinants of anemia among pregnant women in North Gondar Zone, Ethiopia, 2025 (n = 308).

Characteristic	Frequency (*n*)	Anemia prevalence (*n*/%)	AOR (95% CI)	*p*
Age group				
15–19	19 (6.2)	2 (10.5%)	1.6 (0.36–9.21)	0.483
20–24	67 (21.8)	4 (6.0%)	1.1 (0.43–3.38)	0.716
25–29	85 (27.6)	3 (3.5%)	1	—
30–34	98 (31.8)	5 (5.1%)	1.3 (0.52–3.63)	0.502
≥ 35	39 (12.7)	3 (7.7%)	1.5 (0.52–4.71)	0.461
Residence				
Urban	121 (39.3)	5 (4.1%)	1	—
Rural	187 (60.7)	12 (6.4%)	1.5 (0.54–4.12)	0.457
Proximity to water bodies				
< 1 km	92 (29.9)	7 (7.6%)	2.8 (0.92–7.61)	0.030∗
1–2 km	121 (39.3)	6 (5.0%)	1.8 (0.67–5.49)	0.254
> 2 km	95 (30.8)	4 (4.2%)	1	—
Use of ITN				
Consistent	119 (38.6)	3 (2.5%)	1	—
Occasional	112 (36.4)	7 (6.3%)	2.3 (0.62–8.83)	0.181
No ITN	77 (25.0)	7 (9.1%)	3.1 (0.91–10.36)	0.041∗
Knowledge about anemia				
High	111 (36.0)	3 (2.7%)	1	—
Moderate	97 (31.5)	5 (5.2%)	1.9 (0.52–7.60)	0.326
Low	100 (32.5)	9 (9.0%)	3.2 (1.01–10.13)	0.043∗
Gravidity				
Primigravida	128 (41.6)	9 (7.0)	3.0 (1.22–7.48)	0.022∗
Secundigravida	113 (36.7)	5 (4.4)	1.4 (0.50–3.87)	0.516
Multigravida	67 (21.7)	3 (4.5)	1	—
Gestational age				
First trimester	55 (17.9)	3 (5.5)	1.1 (0.32–3.94)	0.851
Second trimester	194 (63.0)	10 (5.2)	1.0 (0.46–2.61)	0.990
Third trimester	59 (19.1)	4 (6.8)	1.3 (0.42–3.95)	0.653

Note: Asterisk “∗” denotes statistically significant p value.

## 4. Discussion

This study explored the prevalence of malaria, its determinants, and its impact on anemia among pregnant women attending ANC in North Gondar Zone, Northern Ethiopia. The findings indicate that malaria remains a persistent public health concern in this population, suggesting that existing control strategies, although effective to some extent, may not be sufficiently reaching high‐risk groups or addressing context‐specific transmission dynamics.

The malaria burden observed in this study is comparable with reports from Ethiopia and other sub‐Saharan African countries [17–22]. This consistency suggests that malaria in pregnancy continues to be a widespread challenge across endemic regions, influenced by variations in ecological conditions, seasonal transmission patterns, and the effectiveness of local intervention programs. Differences observed across studies may not only reflect geographical variability but also methodological differences, including diagnostic techniques and timing of data collection. In contrast, lower prevalence reported in areas such as Boset District [23] highlights how reduced transmission settings and effective control measures can significantly limit malaria risk. The relatively higher burden observed in North Gondar Zone compared with some low‐transmission settings underscores the need for location‐specific strategies tailored to persistent transmission areas [24].

The predominance of P. falciparum is consistent with previous findings in Ethiopia [25, 26]. This has important clinical implications, as P. falciparum is known to cause more severe disease outcomes, particularly in pregnant women, including higher risks of anemia and adverse fetal effects. Its continued dominance reinforces the importance of prioritizing early detection and effective case management within ANC services.

Sociodemographic factors such as rural residence and low educational status were significantly associated with malaria infection. These findings reflect broader structural and behavioral determinants of health, where rural populations often experience increased exposure to mosquito breeding sites and face barriers in accessing healthcare services. Similarly, lower educational levels may limit awareness and reduce the adoption of preventive measures such as consistent use of ITNs [27]. These results highlight the need for targeted health education and community‐based interventions to reduce disparities in malaria risk.

Interestingly, variables such as income, marital status, gravidity, and gestational age were not significantly associated with malaria infection, which is in agreement with findings from other malaria‐endemic settings [28]. This suggests that environmental exposure and behavioral practices may play a more critical role than socioeconomic or obstetric factors in determining malaria risk in this context. Therefore, interventions focusing on improving preventive behaviors may yield greater impact than those addressing economic factors alone.

Environmental and behavioral factors were strongly linked to malaria risk. Living near water bodies, poor housing conditions, and agricultural land use have been widely reported as key contributors to malaria transmission [29–31]. These factors likely increase human–vector contact by providing favorable breeding and resting conditions for mosquitoes. The strong protective effect of consistent ITN use observed in this study aligns with previous evidence [32, 33], reinforcing the importance of sustained behavioral adherence. Together, these findings emphasize that effective malaria control requires an integrated approach that combines environmental management with individual preventive practices.

The study also demonstrated a clear association between malaria infection and anemia. This relationship is biologically plausible and well documented, as malaria contributes to anemia through mechanisms such as hemolysis of infected red blood cells, destruction of uninfected erythrocytes, and suppression of erythropoiesis [34]. In pregnant women, these effects are compounded by increased physiological demands for iron, making them particularly vulnerable. The findings, therefore, highlight the importance of integrating malaria prevention and anemia management within routine ANC services.

Although the prevalence of anemia observed in this study was lower than reported in other Ethiopian studies [35, 36], this difference should be interpreted cautiously. It may reflect variations in study populations, improved access to ANC services, or differences in nutritional status and seasonal timing of data collection. However, it may also indicate potential underestimation or contextual differences that warrant further investigation. The higher occurrence of anemia among specific groups, such as primigravida women and those with limited knowledge, is consistent with previous findings [37]. This suggests that first‐time mothers and less‐informed women represent particularly vulnerable groups and should be prioritized in intervention programs.

From a public health and policy perspective, these findings have important implications. Strengthening ANC services to provide integrated screening and management for both malaria and anemia, improving access to and consistent use of ITNs, and enhancing community awareness, especially in rural areas, could substantially reduce the burden of these conditions. Additionally, addressing environmental risk factors through vector control and improved housing conditions may further enhance prevention efforts.

This study has several strengths, including the use of laboratory‐confirmed malaria diagnosis and hemoglobin measurement, which improve the reliability of the findings. However, some limitations should be considered when interpreting the results. The cross‐sectional design limits causal inference, and seasonal variation may influence malaria prevalence. In addition, reliance on self‐reported data for some variables may introduce recall bias. Therefore, future longitudinal studies are recommended to better understand causal relationships and seasonal dynamics of malaria and anemia in pregnancy.

## 5. Conclusions

Malaria remains a significant public health concern among pregnant women in North Gondar Zone, Northern Ethiopia, with P. falciparum being the predominant species. Environmental exposure, such as living near water bodies, poor housing conditions, and seasonal factors, along with behavioral practices like inconsistent use of ITNs, were identified as key determinants of infection. Malaria infection contributed substantially to maternal anemia, particularly among primigravida women, those with low awareness, and women residing close to water bodies.

These findings highlight the need for strengthened and targeted malaria prevention strategies, including consistent ITN use, improved housing infrastructure, routine malaria screening, and integrated health education during ANC visits. Prioritizing high‐risk groups and reinforcing ANC‐based interventions can help reduce both malaria prevalence and anemia among pregnant women, ultimately improving maternal and fetal health outcomes in endemic regions.

## Author Contributions

G.A.A., T.E., and G.Y. contributed to the conceptualization and design of the study. G.A.A performed data extraction and analysis. G.A.A., T.E., and G.Y. interpreted the results and critically reviewed the manuscript.

## Funding

No funding was received for this manuscript.

## Ethics Statement

The study protocol was reviewed and approved by the Ethics Committee of Debark University (Ref. No: REC/DKU/055/2025). Written informed consent was obtained from all participants prior to enrollment. Participants were clearly informed about the study objectives, procedures, potential risks, and benefits, and their participation was entirely voluntary.

## Conflicts of Interest

The authors declare no conflicts of interest.

## General Statement

The study included pregnant women aged 18 years and above; however, when participants under 18 were encountered, informed assent was obtained from the participant and written consent from a parent or legal guardian, in accordance with ethical guidelines for research involving minors.

Confidentiality was strictly maintained, and each participant was assigned a unique identification code instead of using personal identifiers. All collected data were securely stored on password‐protected computers, and hard‐copy documents were kept in locked cabinets. Access to the data was limited only to the principal investigator and authorized members of the research team.

Written informed consent was documented using signed consent forms, which were securely stored separately from the data to maintain anonymity. Pregnant women diagnosed with malaria or anemia during the study were referred for appropriate treatment in accordance with national guidelines.

## Data Availability

The datasets used and/or analyzed during the current study are available from the corresponding author on reasonable request.
